# Co-expression of mesothelin and CA125/MUC16 is a prognostic factor for breast cancer, especially in luminal-type breast cancer patients

**DOI:** 10.1186/s40364-021-00335-3

**Published:** 2021-10-29

**Authors:** Takahiro Einama, Yoji Yamagishi, Yasuhiro Takihata, Takafumi Suzuki, Tamio Yamasaki, Yuichi Hirose, Kazuki Kobayashi, Naoto Yonamine, Ibuki Fujinuma, Takazumi Tsunenari, Makiko Koga, Yusuke Ishibashi, Ken Nagata, Takehiro Shiraishi, Akiko Nakazawa, Toshimitsu Iwasaki, Eiji Shinto, Kimi Kato, Kimiya Sato, Hideki Ueno, Yoji Kishi, Hitoshi Tsuda

**Affiliations:** 1 Department of Surgery, National Defense Medical College, 3-2 Namiki, 359-8513 Tokorozawa, Saitama Japan; 2 Department of Surgery Basic Pathology, National Defense Medical College, 3-2 Namiki, 359-8513 Tokorozawa, Saitama Japan

**Keywords:** Breast cancer, Mesothelin, CA125/MUC16, Co-expression

## Abstract

The expression of mesothelin correlates with a poor prognosis in patients with breast cancer. Since mesothelin plays a role in cancer metastasis in association with CA125, we herein examined the expression of mesothelin and CA125, and the clinicopathological meaning and prognosis of the co-expression of mesothelin and CA125 in breast cancer. Our results showed that among 478 patients, mesothelin and CA125 were co-expressed in 48 (10 %), mesothelin only in 75 (16 %), CA125 only in 217 (45 %), and neither in 234 (49 %). A high correlation was observed between the expression of mesothelin and CA125 (*P* =0.0004). The co-expression of mesothelin and CA125 correlated with poor patient relapse-free survival (RFS) (*P* = 0.0001) and was identified as an independent predictor of RFS by Cox’s multivariate analysis. In conclusion, this is the first to report the prognostic significance of the co-expression of mesothelin and CA125 in breast cancer. The co-expression of mesothelin and CA125 may be clinically useful for prognostication after surgical therapy in patients with breast cancer.

**Supplementary Information:**

The online version contains supplementary material available at 10.1186/s40364-021-00335-3.

To the Editor:

Mesothelin (MSLN) is a 40-kDa cell surface glycoprotein and expressed not only in normal mesothelial cells slightly [1, 2], but also in various types of cancers [3–6]. Previously, we demonstrated that high MSLN expression was correlated with poor prognosis in breast cancer [7]. CA125/MUC16 (CA125) is one of the binding partners for MSLN [8–11]. Heterotypic adhesion between MSLN and CA125 may cause intracavitary tumor metastasis [8, 10]. We showed co-expression of MSLN and CA125 (Co-expression) were correlated with poor prognosis in pancreatic cancer [11]. However, there have not been any studies regarding Co-expression in breast cancer. Therefore, we investigated CA125 expression in addition to MSLN in breast cancer by immunohistochemistry and examined its association between their co-expression and clinicopathological factors.

Subjects comprised 478 patients who underwent surgical resection for primary breast cancer from January 2002 and December 2013. The clinicopathological parameters of these cases were summarized in Table S1. The immunohistochemical staining and evaluation of mesothelin and CA125 were performed as previously described [11] (Methods S1). The expression of MSLN and CA125 was positive when immunoreactivity was observed in 1 % or more of tumor cells, and negative when immunoreactivity was detected in less than 1 % of cancer cells or was absent. Co-expression was positive when the expression of both MSLN and CA125 was detected, and was negative when the expression of MSLN, CA125, or both was absent (Fig. 1 A).

**Fig. 1:**
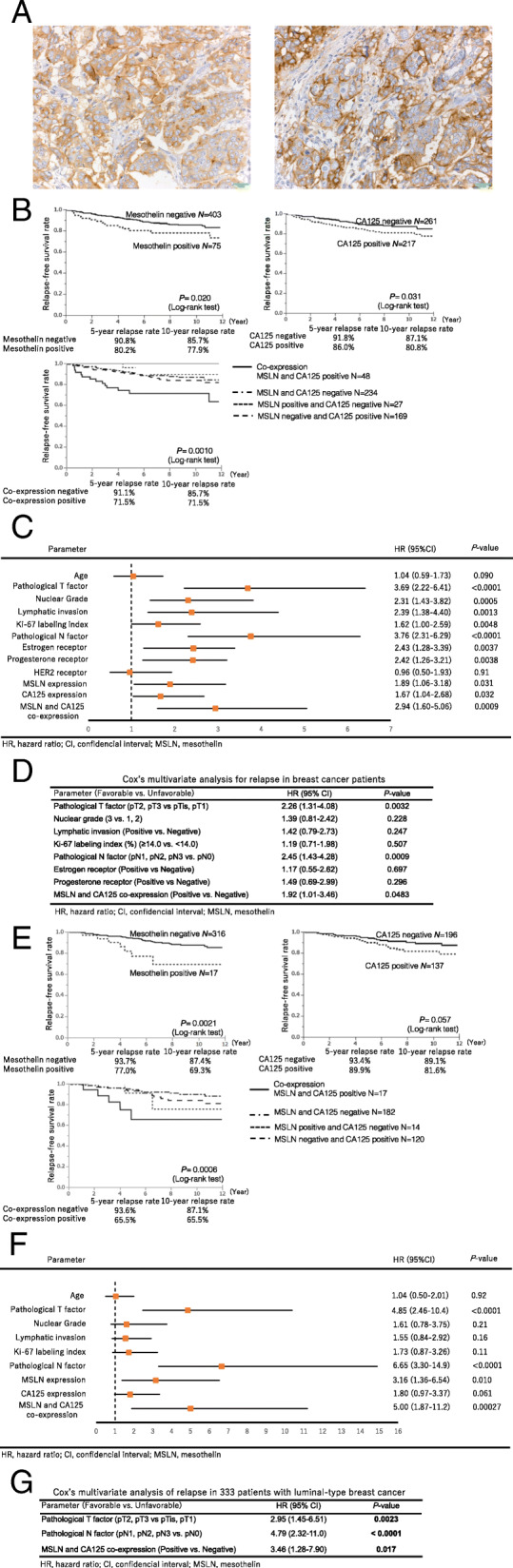
**A** A representative cases of breast cancer is that mesothelin (**A**) and CA125 (**B**) is diffusely positive in triple-negative breast cancer. Immunoperoxidase stain, original magnification ×400. **B** Relapse-free survival curves for 478 patients with breast cancer after surgery classified with the status of the expressions of mesothelin and CA125. Their co-expression group shows the worst prognosis. **C-D** The result of Cox’s univariate analysis is shown in forest plots, and by the Cox’s multivariate analysis, NSLN and CA125 co-expression remains as an independent prognostic factor. **E** Relapse-free survival curves for 333 luminal-type breast cancer patients after surgery classified with the status of the expressions of mesothelin and CA125. Their co-expression group shows the worst prognosis. **F-G** The result of Cox’s univariate analysis was shown in forest plots, and by the Cox’s multivariate analysis, NSLN and CA125 co-expression remains as an independent prognostic factor.

The expression of MSLN was positive in carcinoma cells in 75 (15.7 %) out of 478 breast cancer specimens, while the expression of CA125 was positive in 217 (45.4 %) out of 478 specimens and in 48 (64.0 %) out of 75 MSLN-positive specimens. The positive expression of MSLN correlated with the pathological T factor, triple-negative subtype, Grade 3, a higher Ki-67 labeling index (LI), and higher relapse rate. The positive expression of CA125 also correlated with the subtype and a higher relapse rate. The Co-expression was observed in 48 cases (10.0 %) and correlated with the pathological T factor, triple-negative subtype, Grade 3, a higher Ki-67 LI, and higher relapse rate (Table 1).

**Table 1:** Clinicopathological parameters according to mesothelin and CA125 expression levels

Parameter	Total *N*=478 (%)	Number of cases (%)								
		MSLN			CA125			MSLN and CA125 co-expression		
		Positive *N*=75 (%)	Negative *N*=403 (%)	*P*-value	Positive *N*=217 (%)	Negative *N*=261 (%)	*P*-value	Positive*N*=48 (%)	Negative *N*=430 (%)	*P*-value
Age, years (mean±SD)		60.3 (±11.6)	58.9 (±11.3)	0.33	57.7(±11.2)	60.2 (±11.4)	**0.012**	59.6(±11.6)	60.3 (±11.3)	0.90
Pathological T factor										
T1 (<2 cm)	264	31 (11.8)	233 (88.2)	**0.030**	125 (47.3)	139 (52.7)	0.054	19 (7.2)	245 (92.8)	**0.049**
T2 (2-5 cm)	194	37 (19.1)	157 (80.9)		87 (44.8)	107 (55.2)		24 (12.4)	170 (87.6)	
T3 (>5 cm)	20	7 (35.0)	13 (65.0)		5 (25.0)	15 (75.0)		5 (25.0)	15 (75.0)	
Pathological N factor										
pN1, pN2, pN3	178	29 (16.3)	149 (83.7)	0.78	79 (44.3)	99 (55.6)	0.73	23 (12.9)	155 (87.1)	0.11
pN0	300	46 (15.3)	254 (84.6)		138 (46.0)	162 (54.0)		25 (8.3)	275 (91.7)	
Pathological Stage										
0-I	201	27 (13.4)	174 (86.6)	0.13	100 (49.8)	101 (50.2)	0.26	16 (8.0)	185 (92.0)	0.12
II	221	34 (15.4)	187 (84.6)		94 (42.5)	127 (57.5)		22 (10.0)	199 (90.0)	
III	56	14 (25.0)	42 (75.0)		23 (41.1)	33 (58.9)		10 (17.9)	46 (82.1)	
Subtype										
ER/PgR+ and HER2-	333	31 (9.3)	302 (90.7)	**<0.0001**	137 (41.1)	196 (58.9)	**0.0028**	17 (5.1)	316 (94.9)	**< 0.0001**
ER/PgR+ and HER2+	30	1 (3.3)	29 (96.7)		15 (50.0)	15 (50.0)		0 (0.0)	30 (100.0)	
HER2+	34	7 (20.6)	27 (79.4)		16 (47.0)	18 (53.0)		4 (11.8)	30 (88.2)	
TNBC	81	36 (44.4)	45 (55.6)		49 (60.5)	32 (39.5)		27 (33.3)	54 (66.7)	
Lymphatic permeation										
Positive	284	46 (16.2)	238 (83.8)	0.71	123 (43.3)	161 (56.6)	0.27	24 (8.5)	260 (91.5)	0.16
Negative	194	29 (14.9)	165 (85.1)		94 (48.5)	100 (51.5)		24 (12.4)	170 (87.6)	
Nuclear grade										
1	117	12 (10.3)	105 (89.7)	**< 0.0001**	55 (47.0)	62 (53.0)	0.22	7 (6.0)	110 (94.0)	**< 0.0001**
2	140	4 (2.9)	136 (97.1)		55 (39.3)	85 (60.7)		3 (2.1)	137 (97.9)	
3	221	59 (42.1)	162 (57.9)		107 (48.4)	114 (51.6)		38 (17.2)	183 (82.8)	
Ki-67 labeling index (%)										
≥14	298	33 (11.1)	265 (88.9)	**0.0004**	134 (45.0)	164 (55.0)	0.81	19 (6.4)	279 (93.6)	**0.0008**
<14	180	42 (23.3)	138 (66.7)		83 (46.1)	97 (53.4)		29 (16.1)	151 (83.9)	
Relapse										
Yes	71	17 (23.9)	54 (76.1)	**0.048**	40 (56.3)	31 (43.7)	**0.045**	15 (21.1)	56 (78.9)	**0.0022**
No	407	58 (14.3)	349 (85.7)		177 (43.5)	230 (56.5)		33 (8.1)	374 (91.9)	

*SD* standard deviation, *ER* Estrogen receptor, *PgR* Progesterone receptor, *HER2* Human epidermal growth factor receptor 2, *TNBC* Triple-negative breast cancer, *MSLN* mesothelin

*X*^2^ test.

Values in bold are significantly different.

The relapse free survival (RFS) rate was significantly poorer in patients expressing MSLN or CA125 than in those not expressing MSLN or CA125 Moreover, the prognosis of the group showing the Co-expression was the worst (Fig. 1 B). Cox’s univariate proportional hazards model analyses identified the pathological T factor, NG, lymphatic invasion, Ki-67 LI, and pathological N factor as significant risk factors for recurrence. Both the expressions of MSLN and CA125 were identified as significant risk factors for recurrence: [hazard ratio (HR) 1.89, 95 % confidence interval (CI) 1.06-3.18, *P* = 0.0313 for MSLN; HR 1.67, 95 %CI=1.04-2.68, *P* = 0.0319 for CA125], while Co-expression was a much stronger risk factor (HR 2.94, 95 %CI 1.60-5.06, *P* = 0.0009) (Fig. 1 C). In Cox’s multivariate analyses, Co-expression was an independent predictor of RFS in breast cancer patients (HR =1 0.92, 95 %CI 1.01-3.46, *P* = 0.0483) as well as the pathological T factor (HR = 2.26, 95 %CI 1.31-4.08, *P* = 0.0032) and pathological N factor (HR = 2.45, 95 %CI 1.43-4.28, *P* = 0.0009) (Fig. 1 D, including MSLN and CA125 analysis Table S2).

In 333 patients with hormone receptor-positive (luminal type) breast cancer, the RFS rate was significantly poorer in patients expressing MSLN than in those not expressing MSLN (*P* = 0.0021). The RFS rate also tended to be lower in patients expressing CA125 than in those not expressing CA125 (*P* = 0.057). The prognosis of the group with Co-expression was the poorest (Fig. 1 E). Cox’s univariate and multivariate analyses were performed on 333 luminal-type cases (Fig. 1 F). The expression of MSLN was identified as a significant risk factor for recurrence (HR 3.16, 95 %CI 1.36-6.54, *P* = 0.010). In luminal-type patients, the expression of CA125 was a marginal risk factor for recurrence (HR = 1.80, 95 %CI 0.97-3.37, *P* = 0.0606); however, Co-expression was identified as a significant risk factor (HR = 5.00, 95 %CI 1.87-11.2, *P* = 0.0027). In the multivariate analysis, Co-expression was independent predictors of RFS in luminal-type breast cancer patients (Fig. 1 G, including MSLN and CA125 analysis Table S3).

In conclusion, we herein reported the clinicopathological significance of the co-expression of MSLN and CA125 in breast cancer, particularly in the luminal type, as an independent prognostic factor.

## Supplementary information

**Additional file 1.**

**Additional file 2.**

## Data Availability

Datasets used and/or analyzed during this study are available from the corresponding author on reasonable request.

## References

[1] Chang K, Pai LH, Pass H, Pogrebniak HW, Tsao MS, Pastan I (1992). Monoclonal antibody K1 reacts with epithelial mesothelioma but not with lung adenocarcinoma. Am J Surg Pathol.

[2] Chang K, Pastan I (1996). Molecular cloning of mesothelin, a differentiation antigen present on mesothelium, mesotheliomas, and ovarian cancers. Proc Natl Acad Sci U S A.

[3] Argani P, Iacobuzio-Donahue C, Ryu B, Rosty C, Goggins M, Wilentz RE (2001). Mesothelin is overexpressed in the vast majority of ductal adenocarcinomas of the pancreas: identification of a new pancreatic cancer marker by serial analysis of gene expression (SAGE). Clinical cancer research: an official journal of the American Association for Cancer Research.

[4] Ordonez NG (2003). Application of mesothelin immunostaining in tumor diagnosis. Am J Surg Pathol.

[5] Ho M, Hassan R, Zhang J, Wang QC, Onda M, Bera T (2005). Humoral immune response to mesothelin in mesothelioma and ovarian cancer patients. Clinical cancer research: an official journal of the American Association for Cancer Research.

[6] Einama T, Homma S, Kamachi H, Kawamata F, Takahashi K, Takahashi N (2012). Luminal membrane expression of mesothelin is a prominent poor prognostic factor for gastric cancer. Br J Cancer.

[7] Suzuki T, Yamagishi Y, Einama T, Koiwai T, Yamasaki T, Fukumura-Koga M (2020). Membrane mesothelin expression positivity is associated with poor clinical outcome of luminal-type breast cancer. Oncology letters.

[8] Gubbels JA, Belisle J, Onda M, Rancourt C, Migneault M, Ho M (2006). Mesothelin-MUC16 binding is a high affinity, N-glycan dependent interaction that facilitates peritoneal metastasis of ovarian tumors. Mol Cancer.

[9] Kaneko O, Gong L, Zhang J, Hansen JK, Hassan R, Lee B (2009). A binding domain on mesothelin for CA125/MUC16. J Biol Chem.

[10] Rump A, Morikawa Y, Tanaka M, Minami S, Umesaki N, Takeuchi M (2004). Binding of ovarian cancer antigen CA125/MUC16 to mesothelin mediates cell adhesion. J Biol Chem.

[11] Einama T, Kamachi H, Nishihara H, Homma S, Kanno H, Takahashi K (2011). Co-expression of mesothelin and CA125 correlates with unfavorable patient outcome in pancreatic ductal adenocarcinoma. Pancreas.

