# Supplementary material for: Co-expression of mesothelin and CA125/MUC16 is a prognostic factor for breast cancer, especially in luminal-type breast cancer patients
Source: Biomark Res. 2021 Oct 29;9:78. doi: 10.1186/s40364-021-00335-3 (PMC8555316; doi:10.1186/s40364-021-00335-3)
Supplement: Supplementary file 1 — Additional file 1. [file 40364_2021_335_MOESM1_ESM.docx]

Methods S1

*Immunohistochemistry*

Formalin-fixed paraffin-embedded tissue blocks from 478 patients were collected from the archives of the Pathology Section, Department of Clinical Laboratories, National Defense Medical College Hospital. Tissue microarrays containing two representative tissue cores with a diameter of 2 mm for each case were constructed using a tissue microarrayer (Azumaya, Tokyo, Japan). Four-micrometer-thick sections were cut from these tissue microarray blocks and mounted on charged glass slides, deparaffinized, and rehydrated through a graded series of ethanol. Dako Target Retrieval Solution pH 9.0 (catalog no. S2368; Dako, Carpinteria, CA, USA) was used for antigen retrieval, and slides were boiled in a pressure cooker (Pascal Pressure Cooker, model S2800; Dako) at 125℃ for 3 minutes. Sections were treated with 0.3% hydrogen peroxide for 5 minutes to block endogenous peroxidase activity. One slide for each section was then incubated with a mouse monoclonal antibody against MSLN (clone 5B2 diluted 1:50; Novocastra, Newcastle Upon Tyne, UK), and the other with a mouse monoclonal antibody against CA125 (clone M11 diluted 1:50; Dako) at room temperature for 30 minutes. They were reacted with a dextran polymer reagent combined with secondary antibodies and peroxidase (Envision/HRP; Dako) at room temperature for 30 minutes. Specific antigen-antibody reactions were visualized with 0.2% diaminobenzidine tetrahydrochloride and hydrogen peroxide. Slides were counterstained with hematoxylin for 10 minutes and then gently rinsed in reagent quality water.

*Immunohistochemical Evaluation*

All assessments were performed on the tumor region of the specimen (×200). Each slide was evaluated independently by 2 observers (T.E., Y.Y.) who were blinded to clinical outcomes. Immunostaining for MSLN and CA125 was assessed for both the staining proportion and intensity of tumor cells in each case. MSLN and CA125 expression levels were measured based on the percentage of cells showing the expression of each molecule as follows: <1%, 1% <10%, 10% <50%, and ≥50%. The intensities of the MSLN and CA125 immunoreactions were evaluated using the following scoring system: 1+, incomplete membrane staining and/or faint or barely perceptible cytoplasmic staining in tumor cells; 2+, the entire circumference of the cell membrane was stained and/or cytoplasmic staining exhibited moderate to strong staining. Cytoplasmic granular staining was also scored as 2+. The expression of MSLN and CA125 was positive when immunoreactivity was observed in 1% or more of tumor cells, irrespective of the intensity of immunoreactions, and negative when immunoreactivity was detected in less than 1% of cancer cells or was absent. Co-expression was positive when the expression of both MSLN and CA125 was detected, and was negative when the expression of MSLN, CA125, or both was absent.
