# Supplementary material for: Co-expression of mesothelin and CA125/MUC16 is a prognostic factor for breast cancer, especially in luminal-type breast cancer patients
Source: Biomark Res. 2021 Oct 29;9:78. doi: 10.1186/s40364-021-00335-3 (PMC8555316; doi:10.1186/s40364-021-00335-3)
Supplement: Supplementary file 2 — Additional file 2. [file 40364_2021_335_MOESM2_ESM.docx]

Table S1 Clinicopathological parameters of 478 patients with breast cancer in the present study

| Parameter |  | N=478 (%) |
| --- | --- | --- |
| Age | ≤50 | 115 (24.2) |
|  | >50 | 363 (75.8) |
| Pathological T factor | pTis | 1(0.2) |
|  | pT1 | 263 (55.0) |
|  | pT2 | 194 (40.6) |
|  | pT3 | 20 (4.2) |
| Pathological N factor | N0 | 300(63.0) |
|  | N1 | 123(25.6) |
|  | N2 | 34(7.0) |
|  | N3 | 21(4.4) |
| Pathological Stage | 0 | 1(0.2) |
|  | I | 200(41.8) |
|  | II | 221(46.2) |
|  | III | 56(11.7) |
| Subtype | ER/PgR+ and HER2- | 333 (70.0) |
|  | ER/PgR+ and HER2+ | 30 (6.1) |
|  | HER2+ | 34 (7.1) |
|  | TNBC | 81 (16.8) |
| Lymphatic invasion | Positive | 284(59.4) |
|  | Negative | 194(40.6) |
| Nuclear grade | 1 | 117(24.5) |
|  | 2 | 140(29.3) |
|  | 3 | 221(46.2) |
| Ki-67 labeling index | ≥14 | 298(62.3) |
|  | <14 | 180(37.7) |
| Recurrence | Yes | 71 (13.0) |
|  | No | 407 (87.0) |
| **Number of patients** | **2002 - 2005** | **182 (38.1)** |
|  | **2006 - 2009** | **129 (27.0)** |
|  | **2010 - 2013** | **167 (34.9)** |

ER, ER, Estrogen receptor;

HER2, Human epidermal growth factor receptor 2;

PgR, Progesterone receptor;

TNBC, Triple-negative breast cancer

Table S2 Cox’s univariate and multivariate analyses for relapse in breast cancer patients

| Parameter  (Favorable vs. Unfavorable) | Univariate | |  | Multivariate | | | | | | | |
| --- | --- | --- | --- | --- | --- | --- | --- | --- | --- | --- | --- |
|  |  |  |  | Including mesothelin expression | |  | Including CA125 expression | |  | Including MSLN and CA125 co-expression | |
|  | Hazard ratio  (95% CI) | *P*-value |  | Hazard ratio  (95% CI) | *P*-value |  | Hazard ratio  (95% CI) | *P*-value |  | Hazard ratio  (95% CI) | *P*-value |
| Age  (>50 vs ≤50) | 1.04  (0.59-1.73) | 0.0897 |  |  |  |  |  |  |  |  |  |
| Pathological T factor  (pT2, pT3 vs pTis, pT1) | 3.69  (2.22-6.41) | **< 0.0001** |  | 2.33  (1.35-4.18) | **0.0020** |  | 2.35  (1.36-4.22) | **0.0020** |  | 2.26  (1.31-4.08) | **0.0032** |
| Nuclear grade  (3 vs. 1, 2) | 2.31  (1.43-3.82) | **0.0005** |  | 1.43  (0.739-2.35) | 0.197 |  | 1.50  (0.892-2.58) | 0.128 |  | 1.39  (0.81-2.42) | 0.228 |
| Lymphatic invasion  (Positive vs. Negative) | 2.39  (1.38-4.40) | **0.0013** |  | 1.34  (0.747-2.56) | 0.197 |  | 1.34  (0.744-2.56) | 0.336 |  | 1.42  (0.79-2.73) | 0.247 |
| Ki-67 labeling index (%)  (≥14.0 vs. <14.0) | 1.62  (1.00-2.59) | **0.0481** |  | 1.25  (0.753-2.07) | 0.380 |  | 1.28  (0.774-2.11) | 0.332 |  | 1.19  (0.71-1.98) | 0.507 |
| Pathological N factor  (pN1, pN2, pN3 vs. pN0) | 3.76  (2.31-6.29) | **< 0.0001** |  | 2.61  (1.54-4.54) | **0.0003** |  | 2.54  (1.50-4.43) | **0.0005** |  | 2.45  (1.43-4.28) | **0.0009** |
| Estrogen receptor  (Positive vs Negative) | 2.43  (1.28-3.39) | **0.0037** |  | 1.26  (0.59-2.83) | 0.548 |  | 1.24  (0.60-2.78) | 0.566 |  | 1.17  (0.55-2.62) | 0.697 |
| Progesterone receptor  (Positive vs Negative) | 2.42  (1.26-3.21) | **0.0038** |  | 1.54  (0.72-3.09) | 0.256 |  | 1.53  (0.71-3.07) | 0.267 |  | 1.49  (0.69-2.99) | 0.296 |
| HER2 receptor  (Negative vs Positive) | 0.96  (0.50-1.93) | 0.91 |  |  |  |  |  |  |  |  |  |
| Mesothelin expression  (Positive vs. Negative) | 1.89  (1.06-3.18) | **0.0313** |  | 1.34  (7.38-2.35) | 0.319 |  |  |  |  |  |  |
| CA125 expression  (Positive vs. Negative) | 1.67  (1.04-2.68) | **0.0319** |  |  |  |  | 1.60  (1.00-2.58) | **0.0494** |  |  |  |
| MSLN and CA125 co-expression  (Positive vs. Negative) | 2.94  (1.60-5.06) | **0.0009** |  |  |  |  |  |  |  | 1.92  (1.01-3.46) | **0.0483** |

CI, confidence interval

Table S3 Cox’s univariate and multivariate analyses of relapse in 333 patients with luminal-type breast cancer

| Parameter  (Favorable vs. Unfavorable) | Univariate | |  | Multivariate | | | | |
| --- | --- | --- | --- | --- | --- | --- | --- | --- |
|  |  |  |  | Including mesothelin expression | |  | Including MSLN and CA125 co-expression | |
|  | Hazard ratio  (95% CI) | *P*-value |  | Hazard ratio  (95% CI) | *P*-value |  | Hazard ratio  (95% CI) | *P*-value |
| Age  (>50 vs ≤50) | 1.04  (0.50-2.01) | 0.919 |  |  |  |  |  |  |
| Pathological T factor  (pT2, pT3 vs pTis, pT1) | 4.85  (2.46-10.4) | **< 0.0001** |  | 2.93  (1.44-6.47) | **0.0027** |  | 2.95  (1.45-6.51) | **0.0023** |
| Nuclear grade  (3 vs. 1, 2) | 1.61  (0.78-3.75) | 0.205 |  |  |  |  |  |  |
| Lymphatic invasion  (Positive vs. Negative) | 1.55  (0.84-2.92) | 0.160 |  |  |  |  |  |  |
| Ki-67 labeling index (%)  (≥14.0 vs. <14.0) | 1.73  (0.87-3.26) | 0.110 |  |  |  |  |  |  |
| Pathological N factor  (pN1, pN2, pN3 vs. pN0) | 6.65  (3.30-14.9) | **< 0.0001** |  | 5.05  (2.43-11.57) | **< 0.0001** |  | 4.79  (2.32-11.0) | **< 0.0001** |
| Mesothelin expression  (Positive vs. Negative) | 3.16  (1.36-6.54) | **0.010** |  | 2.96  (1.25-6.26) | **0.016** |  |  |  |
| CA125 expression  (Positive vs. Negative) | 1.80  (0.97-3.37) | 0.061 |  |  |  |  |  |  |
| MSLN and CA125 co-expression  (Positive vs. Negative) | 5.00  (1.87-11.2) | **0.0027** |  |  |  |  | 3.46  (1.28-7.90) | **0.017** |

CI, confidence interval
